# Mentoring future mentors in undergraduate medical education

**DOI:** 10.1371/journal.pone.0273358

**Published:** 2022-09-15

**Authors:** Yun Ting Ong, Chrystie Wan Ning Quek, Anushka Pisupati, Eleanor Kei Ying Loh, Vaishnavi Venktaramana, Min Chiam, Lalit Kumar Radha Krishna

**Affiliations:** 1 Yong Loo Lin School of Medicine, National University of Singapore, Singapore, Singapore; 2 Division of Supportive and Palliative Care, National Cancer Centre Singapore, Singapore, Singapore; 3 Division of Cancer Education, National Cancer Centre Singapore, Singapore, Singapore; 4 Duke-NUS Medical School, Singapore, Singapore; 5 Palliative Care Institute Liverpool, Academic Palliative & End of Life Care Centre, University of Liverpool, Liverpool, United Kingdom; 6 Cancer Research Centre, University of Liverpool, Liverpool, United Kingdom; 7 Centre of Biomedical Ethics, National University of Singapore, Singapore, Singapore; 8 PalC, The Palliative Care Centre for Excellence in Research and Education, Singapore, Singapore; Bilawal Medical College, Liaquat University of Medical and Health Sciences, PAKISTAN

## Abstract

**Background:**

Efforts to support flagging mentoring programs facing shortages of experienced clinical mentors have had an unexpected and welcome effect. Supplementing traditional mentoring programs with peer-mentoring have not only addressed gaps in practice, structure, support and mentee oversight but have offered mentees charged with peer-mentoring duties the opportunity to take on mentoring roles under senior supervision. This study evaluates the experiences of peer-mentors within a local research mentoring program to better understand and advance this endeavor.

**Methods:**

Semi-structured interviews and post-interview surveys based on recent reviews on mentoring were employed. Adapting the Systematic Evidence Based Approach, data was analysed using thematic and content analysis. Results were combined using the Jigsaw Perspective to ensure that key elements of the different mentoring stages were identified.

**Results:**

The interviews and surveys revealed the following domains: Motivation, Initiation, Practicing, and Mentoring Environment.

**Conclusion:**

These findings provide novel insight into a structured framework that may help guide the experiences, training, assessment, and oversight of peer-mentors beyond the auspices of our local program. These general observations will equip host organizations with the direction they need to take in designing and executing peer-mentoring training and assessment programs of their own. Whilst the stages of peer-mentoring need further evaluation and an effective means of assessment and support pivotal, we believe our findings suggest that peer-mentoring may not only help to address the shortfall in mentors but is an invaluable learning experience that prepares and instils key values, beliefs and principles in young would-be mentors.

## Introduction

A shortage of experienced clinical mentors [1], changing mentoring and clinical practice, and shifting expectations upon mentees, mentors and mentoring relationships particularly during the COVID-19 pandemic [2–5] has seen mentoring programs struggle [6]. Traditional concepts of dyadic relationships between an experienced and trained physician and a medical student or junior doctor have come under scrutiny over their ability to contend with raising concerns over gaps in structure [7, 8], assessment and oversight [9] of mentoring relationships and the mentoring environment [10, 11]. Indeed, recent reviews of ethical issues in mentoring suggest that inadequate structuring of the mentoring process invites the possibility of bullying, racism, sexism and the misappropriation of the mentee’s work [12–14]. This, in turn, threatens mentoring’s central objective which is to advance the mentee’s development, goals and interests.

As a result, some programs have supplemented their traditional mentoring approaches with electronic-mentoring (e-mentoring) [15], peer-mentoring [16, 17] and group-mentoring [18–21] to better align expectations between mentees and mentors, to introduce and police codes of practice and to enhance personalized, appropriate, specific, timely, holistic and longitudinal support.

### The palliative medicine initiative

Acknowledging these concerns, the Palliative Medicine Initiative (PMI), a research mentoring program at the Divisions of Supportive and Palliative Care (DSPC) and Cancer Education (DCE) at the National Cancer Centre Singapore (NCCS), adopted a Combined novice, e-mentoring and peer-mentoring (CNEP) approach. *(S1 Appendix. Definition of Novice Mentoring, E-Mentoring and Peer-Mentoring).* This was to supplement its mentor-depleted novice mentoring program [22].

Whilst the PMI has supported more than 100 single-authored, mentee co-authored and/or mentee first-authored publications in peer-reviewed journals, and more than 150 posters at international conferences in palliative medicine, medical ethics, medical education, end-of-life ethics and health services research over the last 12 years, its novice mentoring approach has been especially susceptible to mentor shortages. To attend to these gaps, the PMI adopted the CNEP approach detailed in Krishna et al. [22]’s study entitled “*Combined novice*, *near-peer*, *e-mentoring palliative medicine program*: *A mixed method study in Singapore*”.

Designed around mentoring reviews [23–25] and fashioned around a research process that includes stages such as mentee initiation, mentee-mentor matching, initial meetings, data gathering, data analysis, manuscript writing, submission and post submission, the CNEP framework provides a structured and consistent stage-based approach that allows mentors and program administrators to assess the progress of peer-mentor’s and mentee’s. Completion of the stages not only provides them with skills and knowledge but presents longitudinal opportunities for assessing their values, attitudes, working styles, intercollegial interactions and ability to adapt to the PMI culture. Mentees who complete all the stages are deemed research competent. Research competent mentees who contribute to the practice, culture, and structure of the PMI are deemed ‘PMI competent’ by the mentors and program administrators. It is ‘PMI competent’ mentees that are invited to take on the role of ‘peer-mentors’. ‘Near-peer mentors’ and ‘peer-mentors’ are used interchangeably here. ‘Mentors’ and ‘senior mentors’ are used interchangeably as well.

Whilst the success of the CNEP approach in enhancing the mentoring experience of mentees have been well established, feedback by peer-mentors have revealed rather surprising findings. Successful PMI mentees report taking on the role of mentor under senior supervision as a means of advancing their mentor training and interests. With two peer-mentors becoming mentors in the PMI, these unexpected findings demand closer scrutiny. The notion that peer-mentoring could be employed to train future mentors is especially appealing not only to sustain the culture and structure of the program but to mould their development around the program’s time-honoured values, beliefs and principles. With no studies we are aware of looking into the effects of peer-mentoring as a means of mentor training, we propose an evaluation of the experiences of peer-mentors in the hope that insights provided will not only enhance the PMI experience but facilitate the design and support of similar programs in other settings.

## Methods

To answer our primary research question, “*how does being a peer-mentor impact a mentee’s development*?” and “*what factors influence a peer-mentor’s development*?”, we adopted a combination of semi-structured individual interviews to capture individual experiences in our peer-mentoring program and post-interview surveys designed using the MEntor Reflection InstrumenT (MERIT) framework [26]. This approach allowed us to explore the participant’s reflections after the interview and investigate their specific mentoring experiences.

### Systematic Evidence Based Approach (SEBA)

**Fig 1:**
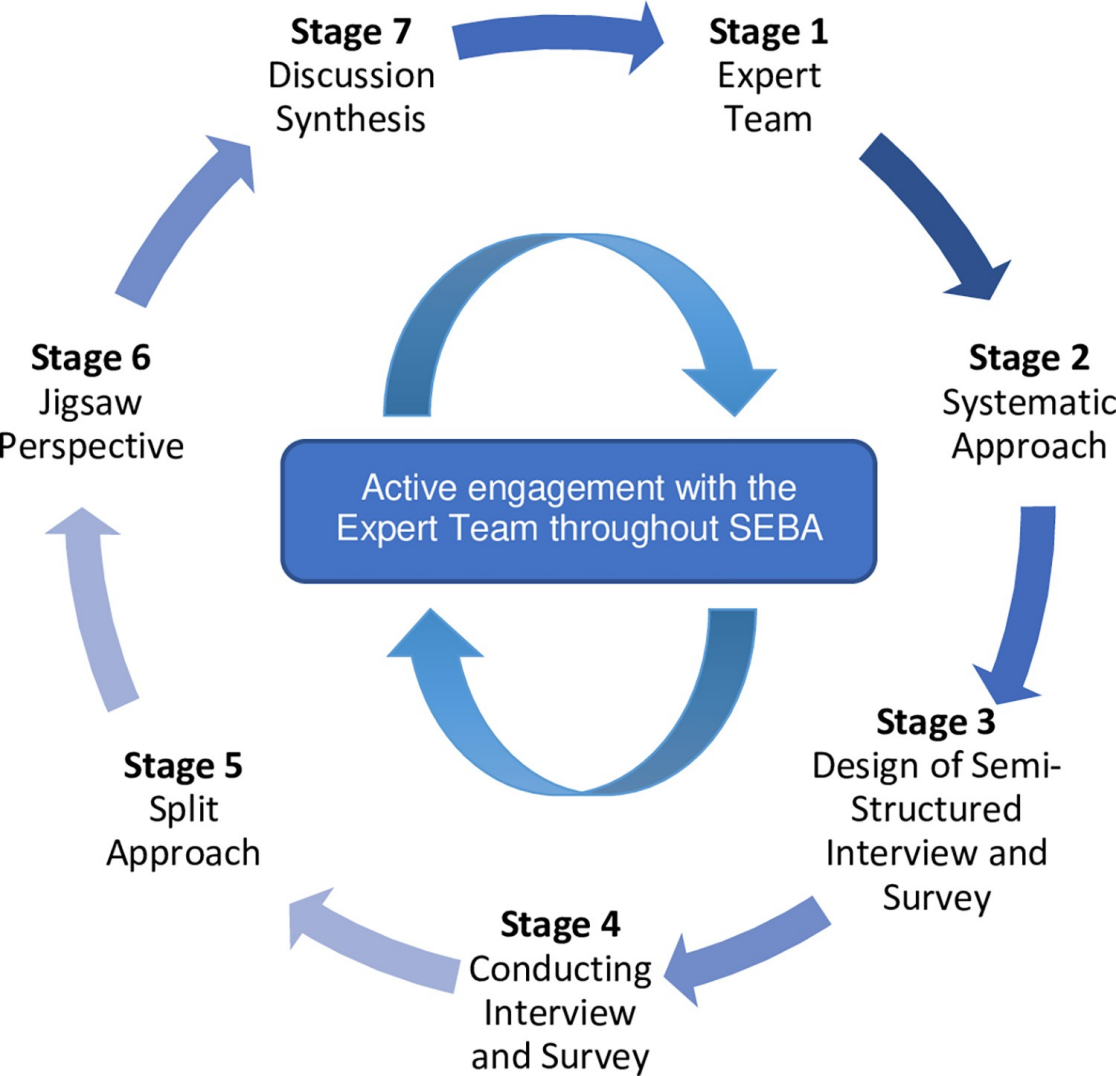
The prospective SEBA process.

The adapted SEBA methodology was used. It comprises the following stages: 1) Expert Team, 2) Systematic Approach, 3) Design of Semi-Structured Interview and Survey, 4) Conducting Interview and Survey 5) Split Approach 6) Jigsaw Perspective and 7) Discussion Synthesis *(Fig 1).*

#### Stage 1. Expert team

An expert team consisting of a medical librarian from the Yong Loo Lin School of Medicine (YLLSoM) at the National University of Singapore (NUS), and local educational experts and clinicians at NCCS, YLLSoM, the Palliative Care Institute Liverpool and Duke-NUS Medical School ensured that the SEBA methodology was employed in a consistent manner within accepted practices. The expert team reviewed the data, its analysis, and provided oversight to ensure a transparent and reproducible process.

#### Stage 2. Systematic approach

Guided by current data, the expert and research teams adopted a systematic approach to review current accounts of peer-mentoring [8, 23, 27] to design a semi-structured interview questionnaire and post-interview survey.

*Ethics Approval and Consent to Participate*. Ethics approval (reference number: 202010–00084 and 202103–00057) was obtained from the SingHealth Combined Institutional Review Board.

#### Stage 3. Design of semi-structured Interview and survey

*Theoretical lens*. With peer-mentoring and CNEP mentoring shown to be sociocultural constructs, the design of the semi-structured interview and survey demanded due consideration of individual, interactional and environmental factors. This saw the adoption of a constructivist ontological position and relativist epistemological perspective consistent with SEBA’s roots. The post-survey was designed to tap on the participant’s reflections triggered by the interviews.

*Instrument*. The interview guide was designed based on data drawn from recent studies of our program [1, 8, 22, 28], reviews of mentoring programs [7, 9, 12, 13, 27], mentoring practice [15, 23], mentoring assessments [11, 14, 16, 17, 24], the mentoring environment [9] and the influence of the host organization [16]. These considerations also paved the way for the adoption and adaptation of the MERIT framework which sits at the heart of the post-interview survey.

#### Stage 4. Conducting semi-structured interview and survey

*Setting and context*. The semi-structured interviews were conducted at DSPC between 8^th^ February 2021 and 13^th^ May 2021 using the Zoom video conferencing platform. The post-interview surveys were conducted on the secure platform Form.gov.sg.

*Subjects*. Peer-mentors in our mentoring program were invited to participate. Purposive sampling was conducted with email invitations sent to eligible peer-mentors containing a participant information sheet which detailed the study’s nature, duration and aims. The e-mail invitations also stressed participant anonymity in the 30-minute audio-recorded interview and the participant’s right to withdraw from the study at any point and without prejudice. All participants provided written and verbal consent. Recruitment stopped when data saturation was reached.

*Study design*. Two trained members of the research team (CQWN and AP) briefed on the study aims conducted the interviews. Audio recordings were transcribed verbatim using the NVivo 12 Software, anonymized with their integrity verified.

#### Stage 5: Split approach

Following the initial review of the thematic analysis, the expert team suggested the use of content analysis to ensure that key elements of peer-mentoring were captured. Concurrent use of Hsieh and Shannon [29]’s approach to directed content analysis and Braun and Clarke [30]’s thematic analysis ensured a comprehensive, reproducible and transparent analysis [31–33].

*Thematic analysis*. Using Braun and Clarke’s approach to thematic analysis, three study team members constructed codes from the surface meaning of the interview transcripts. Their independent findings were discussed at online meetings. Sandelowski and Barroso [34]’s ‘negotiated consensual validation’ was used to achieve consensus and to establish a common coding framework and code book. Subthemes and themes were developed upon collapsing the codes and larger inclusive concepts into even larger groups. A memo of reflexivity was kept for documentation.

*Directed content analysis*. Using Hsieh and Shannon’s approach to directed content analysis, two additional study team members extracted categories drawn from key publications on mentoring stages and the CNEP approach. These were Krishna et al. [1]’s study entitled *Mentoring stages*: *A study of undergraduate mentoring in palliative medicine in Singapore* and Krishna et al. [22]’s study entitled *Combined novice*, *near-peer*, *e-mentoring palliative medicine program*: *A mixed method study in Singapore*.

Consensus on the final themes and subthemes, and categories and subcategories, were then discussed and agreed upon across both groups.

#### Stage 6 of SEBA: Jigsaw perspective

Similarities and/or areas of overlap between the themes and categories allowed them to be combined like pieces of a jigsaw puzzle. The combined themes and categories are referred to as domains.

To guide the melding of similar themes and categories, and to maintain a sense of transparency, the Jigsaw Perspective was guided by Phases 4 to 6 of France et al. [35]’s adaptation of Noblit and Hare [36]’s seven phases of meta-ethnography. The themes and categories that are to be combined undergo reciprocal translation to determine if the respective themes and categories truly reflect one another. To do so, the codes and subcategories and/or subthemes within respective articles were reviewed.

Study of the initial results underlined the need for contextualized data. As a result, we have included more details of the PMI CNEP program at the start of the study and introduced contextualized data in the results and discussion sections.

## Results

All 12 of the invited peer-mentors participated in the interviews and 11 completed the post-interview survey.

### A. Semi-structured interviews

The domains yielded from the synthesis of the Split Approach are: **1) Motivation, 2) Initiation, 3) Practicing, and 4) The Mentoring Environment.** Table 1 shows the demographic of the peer-mentors interviewed.

**Table 1:** Demographic of interviewees.

	Peer’s Role	Number of Peer-Mentored Projects	Approximate Duration
1	Junior Doctor	At least 2 projects	3 years
2	Junior Doctor	2 projects	3 years
3	Junior Doctor	At least 3 projects	4 years
4	Medical Student, M4	3 projects	3 years
5	Medical Student, M4	At least 2 projects	3 years
6	Medical Student, M4	At least 3 projects	2.5 years
7	Junior Doctor	At least 3 projects	2 years
8	Medical Student, M3	1^st^ project- submitted	1 year
9	Medical Student, M4	1^st^ project- submitted	1.5 years
10	Medical Student, M2	1^st^ project- data analysis	1 year
11	Medical Student, M4	1^st^ project- published	2 years
12	Medical Student, M4	1^st^ project- published	2 years

#### Domain 1: Motivation

Eight of the twelve (66.67%) participants were motivated to take on the role of a peer-mentor to develop their leadership skills.

*“It helps you to grow as a student or as a leader. …this enticed me to be a near-peer mentor.” (peer-mentor 7)*

Eight of the twelve (66.67%) participants were motivated to ‘pay it forward’.

*“…also inspired me to agree to be a near-peer mentor myself was seeing the impact of my own senior mentors…” (peer-mentor 2)*

#### Domain 2: Initiation

Taking on the role of a peer-mentor involves practical and personal considerations. From a personal perspective, participants reported ‘first day anxieties’ (n = 8, 66.67%).

*"The biggest problem when approaching peer mentorship for me is trying to have the confidence to actually teach the skills necessary… it’s also my first time doing a lot of things and I didn’t want to teach the wrong thing." (peer-mentor 10)*

Participants also reported the need to reframe their individualistic perspectives to achieve common goals (n = 10, 88.33%).

*"The perspective change, from…focusing on myself, to focus[ing] on others." (peer-mentor 3)*

Practical perspective-taking on this new position required an alignment of expectations and an understanding of their roles and responsibilities. All twelve participants reported being introduced to the mentoring approach and discussing their roles and responsibilities. For nine participants (75%), their concerns required individual discussions with their mentor or senior peer-mentor.

*“She explained whatever I had to do and said that I could ask her for help whenever I need[ed] to or had any clarifications. So, I think that helped to allay my fears." (peer-mentor 8)*

#### Domain 3: Practicing

A peer-mentor’s role brought with it a deeper appreciation of the mentoring approach and the impact of the mentoring environment upon their experiences. The role of peer-mentor also brought a greater sense of independence (n = 12, 100%) and reaffirmed the peer-mentor’s values and beliefs (n = 12, 100%).

*"This idea of being generous or paying it forward or giving back to people [was] reinforced during my process as a peer-mentor." (peer-mentor 3)*

Peer-mentors also reported enhanced leadership (n = 10, 83.33%), reflective (n = 12, 100%), communication (n = 12, 100%) and active listening (n = 8, 66.67%) skills as well as being better at nurturing relationships (n = 11, 91.67%) and role modelling (n = 12, 100%). Peer-mentors also reported being more adaptable, accountable to their mentees and mentors, approachable, collaborative, confident, empathetic, open-minded, humble, meticulous, patient, resilient, self-aware, trustworthy and independent.

#### Domain 4: The mentoring environment

Participants reported that the mentoring program’s structure and culture influenced their development. Peer-mentors attributed their overall development to the program’s consistent structure (n = 12, 100%), clarity on roles and job scopes (n = 11, 91.67%) and the presence of online platforms (n = 11, 91.67%). Similarly, the mentoring program’s supportive culture (n = 12, 100%) and duty to respect each other (n = 12, 100%) helped them mature as mentors. These changes were also facilitated by the mentor’s guidance and support (n = 11, 91.67%) and their approachable, available and proactive nature (n = 10, 83.33%)

### B. Post-interview surveys

The findings of the post-interview survey (n = 11, with 1 non-response) some 3–4 weeks after the initial interviews reflected similar findings *(S2 Appendix. Post-interview Survey Questions)*. The results re-iterated the notion that peer-mentors were motivated to provide research, career and academic guidance and facilitate mentee development out of a desire to ‘pay it forward’ and help others.

Peer-mentors also believed that their mentoring approach was guided by a combination of their previous experiences in the program, the presence of role modelling, regnant values, professional expectations and the mentoring approach and culture.

## Discussion

### Stage 7 of SEBA: Discussion synthesis

In keeping with the SEBA methodology, the initial results were reviewed by the expert and research teams. This process revealed that, without appropriate information on the structuring and practice of the PMI CNEP program, much of the nuances surrounding the findings would be lost. As a result, the discussion section will include information on the structure, culture, and environment of the PMI’s practice.

### Peer-mentoring stages

The data accrued here suggests the presence of mentoring stages. This finding is not surprising given earlier reports of mentee ‘mentoring stages’ [1, 22] derived from the PMI’s mentor-guided, research-based framework [8, 9].

**Fig 2:**
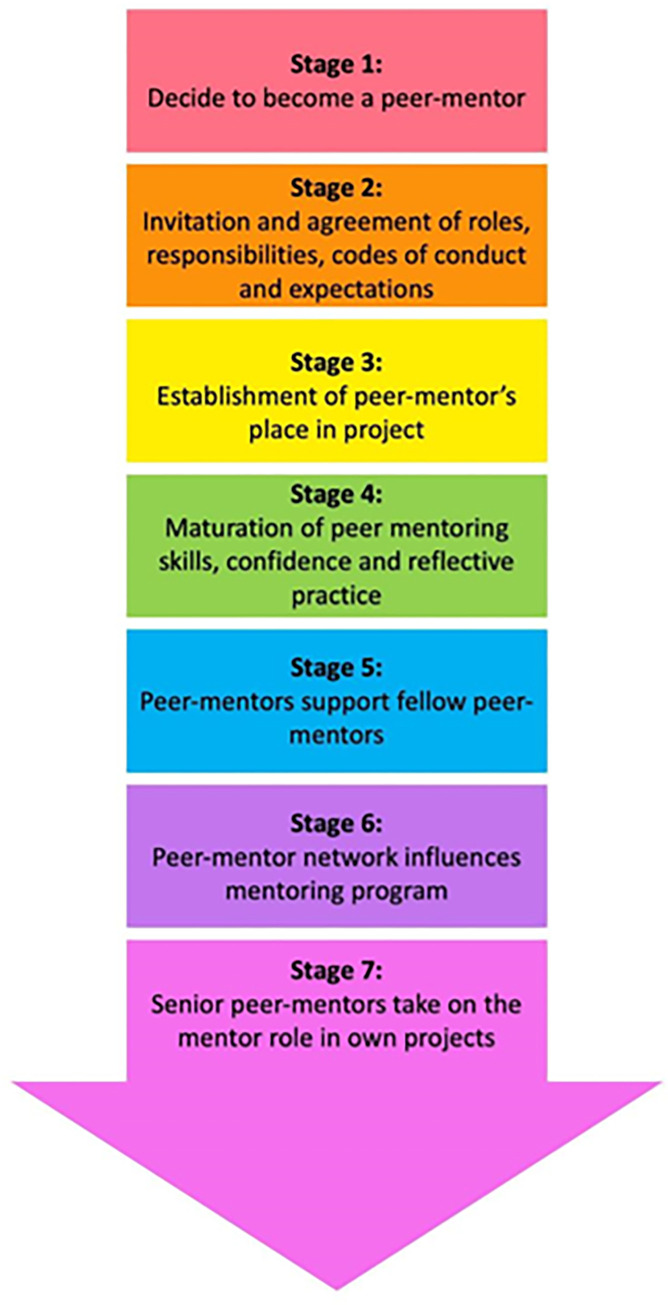
Peer-mentor development stages.

As shown in Fig 2, Stage 1 begins with an invitation to become a peer-mentor. Stage one is drawn from Domain 1 (Motivation) and foregrounded by the fact that only ‘PMI competent’ mentees are invited. This selection process ensures a comprehensive review of the potential peer-mentor’s abilities, availabilities and motivations. It ensures that selections are not biased and that vulnerable mentees are not pressured into participating.

Enlisting ‘PMI competent’ mentees provide an indication as to whether prospective peer-mentors will find the roles, responsibilities and codes of conduct expected of peer-mentors acceptable. In addition, the personalized invitation and the offer to collaborate helps to build the mentee’s confidence. It provides them with the assurance that their efforts, competencies and potential are recognized and appreciated. It also provides motivated ‘PMI competent’ mentees a chance to build on their skills and knowledge within a working environment and learning structure that they are already aware of and are comfortable with. Aside from the chance to lead a team on a project that they are likely to be interested in with a mentor that they have worked with in the past, the invitation to be a peer-mentor also provides them with a chance to improve practice, culture, and structure of the PMI. The prospective peer-mentor is then given time to consider the offer and discuss their queries with the mentor before accepting the invitation.

Stage 2 is based on Domains 1 (Motivation) and 2 (Initiation). It begins with acceptance of the invitation and an agreement of roles, responsibilities, codes of conduct and expectations associated with the peer-mentoring role. This stage can be difficult for new peer-mentors, and they often rely upon their mentors or senior peer-mentors for guidance and support.

Drawing on regnant CNEP practice to contextualize the data garnered in Domain 2 (Initiation), this stage sees new peer-mentors build on their ‘cognitive base’ and adopt appropriate time management and self-management strategies to contend with the additional work. The mentors should provide additional resources and training to prepare the peer-mentor for their new role and responsibilities. Timelines, expectations and the approach adopted for the specific mentoring project are also discussed and agreed upon. As highlighted in Domain 2 (Initiation), this stage also represents a key opportunity to agree upon lines of communication and reiterate codes of conduct, expected assessment outcomes, feedback loops, methods used and support mechanisms available. These processes underscore the personalized nature of recruitment and support of a peer-mentor. It also marks the beginning of a new mentoring relationship between the mentor and peer-mentor.

Stage 3 sees the establishment of the peer-mentor’s place within the mentoring project. Drawing on Domain 3 (Practicing), this stage sees peer-mentors orientated to their roles and introduced to the mentees. This stage proceeds with mentors and peer-mentors working together to create an open and supportive mentoring environment that allows open discussion and trusting relationships between mentees, peer-mentors and the mentor. These personalized relationships provide them with the opportunity to hone their listening, communication, leadership, empathetic and reflective skills under senior supervision.

Stage 4 sees the maturation of peer-mentoring skills, growing confidence, and the development of deeper reflective practice on the part of the peer-mentor. Drawing from current CNEP practice, we see peer-mentors acquire a more nuanced understanding of teamwork, the mentoring process, the relationships with the people around them and the mentoring environment. This stage brings personal and professional development for the peer-mentor. Having previously experienced the mentee’s role, trajectory and evolving needs, it is notable that peer-mentors will likely begin to exhibit desirable mentoring traits (6, 9, 10, 27). Drawing on Domains 3 (Practice) and 4 (Mentoring Environment), Stage 4 is marked by the peer-mentor’s growing confidence. This is exemplified by greater adaptability, patience, approachability, open-mindedness, empathy, collaborativeness and self-awareness which are key professional characteristics (6, 9, 10, 27).

Domain 4 (Mentoring Environment) also provides a glimpse of the support provided for professional and personal development within the CNEP program. This includes role modelling and guidance by the mentor, and guided reflections, peer-mentoring experiences, interactions and mentee feedback. In addition, Domain 4 (Mentoring Environment) reveals the impact of the mentoring structure in shepherding practice, the effects of the mentoring approach on steering practice and interactions, and the mentoring culture that focuses on ‘paying it forward’ and supporting one another. The post-interview surveys reveal that the peer-mentor’s own reflections and insights personalize the effects of the structure, approach and culture within the mentoring environment.

Stage 5 also draws on Domain 4 (Mentoring Environment). It sees peer-mentors using their experiences to help their fellow peer-mentors. The data reveals the organic formation of a support system where more experienced and senior peer-mentors guide and share information with more junior peer-mentors.

Drawing on the post-interview surveys, Stage 6 sees the peer-mentoring network begin to influence the wider mentoring program. This is primarily by shaping existing mentoring codes of practice, the program’s culture and expectations, and its hidden curriculum.

**Fig 3:**
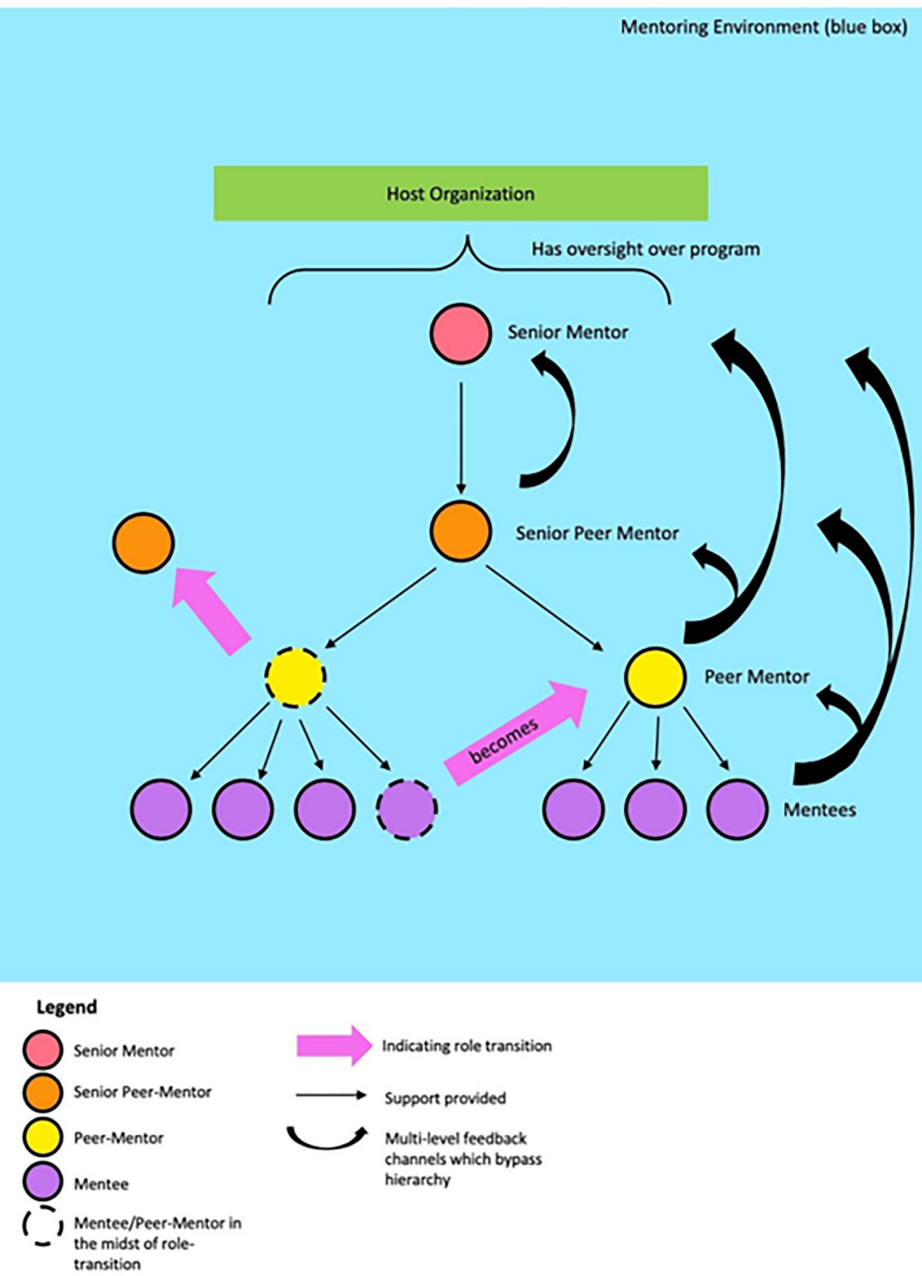
Overview of the mentoring program.

Stage 7 draws on current CNEP practice and focuses on the more senior peer-mentor taking on the mentor’s role in their own projects. Stages 6 and 7 serve to underline the importance of longitudinal and holistic review of the mentoring relationship and the program as a whole (Fig 3). In doing so, it underscores the need for effective assessment tools to evaluate individual mentoring relationships and the wider mentoring program.

We believe this framework will help guide and structure the experiences, training, assessment, and oversight of peer-mentors beyond the auspices of the PMI. These general observations may equip host organizations with the direction they need to take in designing and executing peer-mentoring training and assessment programs of their own.

## Limitations

This study is not without its limitations. Participants in this study were represented by 12 peer-mentors in a single centre research-based mentoring program for medical students and junior doctors in Singapore. This setting and the unique approach adopted by the mentoring program may raise some questions as to the applicability of these findings outside the program and in other clinical settings.

Additionally, as peer-mentors were interviewed at a single time point, often after completion of a project or in the later stages of their research process, recall bias and the halo effect may surface.

Suggestions of peer-mentoring reducing the presence of hierarchy within programs and thus facilitating more open and honest exchange of ideas may also be compromised given the presence of strict levels of seniority in medical school and the practice of medicine. This may lead to the possibility of social desirability bias amongst responders.

## Conclusion

In forwarding these new insights, this study underscores the critical responsibility of mentors and the host organization in nurturing peer-mentors–particularly evident in their role in recruiting, training, overseeing and enabling a supportive and safe environment for mentees, peer-mentors and mentors alike. Whilst the stages of peer-mentoring need further evaluation and an effective means of assessment and support pivotal, we believe our findings suggest that peer-mentoring may not only help to address the shortfall in mentors but is an invaluable learning experience that prepares and instils key values, beliefs and principles in mentees with greater mentoring aspirations. The impact of these experiences on their professional identity formation ought to be the focus of future study.

## Supporting information

S1 Appendix Definition of novice mentoring, e-mentoring and peer-mentoring. (DOCX)

S2 Appendix Post-interview survey questions. (DOCX)
