# Supplementary material for: Mentoring future mentors in undergraduate medical education
Source: PLoS One. 2022 Sep 15;17(9):e0273358. doi: 10.1371/journal.pone.0273358 (PMC9477267; doi:10.1371/journal.pone.0273358)
Supplement: S1 Appendix — (DOCX) [file pone.0273358.s001.docx]

**SUPPLEMENTARY INFORMATION**

**S1 Appendix. Definition of Novice Mentoring, E-Mentoring and Peer-Mentoring**

**Novice mentoring** adopted by the PMI is characterized as a “*dynamic*, *context dependent*, *goal sensitive*, *mutually beneficial relationship between an experienced clinician (mentor) and junior clinicians and/or under- graduates (mentee) that is focused upon advancing the development of the mentee*” [1].

**E-mentoring** is defined as a “*personalized, internet or electronically mediated approach that is largely used to complement face-to-face mentoring to provide personalized, appropriate, specific, timely, holistic, accessible and longitudinal mentoring support to build mutually beneficial mentoring relationships between the host organization, a senior mentor and an individual mentee. Working within the confines of prevailing professional codes of conduct and standards of practice this approach is focused upon realizing the goals and needs of the mentee, the mentor, the host organization that supports and oversees the program and their relationships. Its asynchronous nature also nurtures reflective practices that helps develop deeper mentoring relationships”* [2].

**Peer-mentoring** is characterized as a “*voluntary collaboration between colleagues of similar rank and experience and common academic interests on mutually beneficial structured fixed term projects. These processes often include a senior clinician who facilitates discussions, provides personalized support and feedback and oversees the mentoring process. Effective peer-mentoring nurtures long-term friendships and professional collaborations between peers*” [3].

**References**

1. Krishna L, Toh Y, Mason S, Kanesvaran R. Mentoring stages: A study of undergraduate mentoring in palliative medicine in Singapore. PloS one. 2019;14(4):e0214643–e. pmid:31017941
2. Chong JY, Ching AH, Renganathan Y, Lim WQ, Toh YP, Mason S, et al. Enhancing mentoring experiences through e-mentoring: a systematic scoping review of e-mentoring programs between 2000 and 2017. Advances in Health Sciences Education. 2019:1–32.
3. Lim SYS, Koh EYH, Tan BJX, Toh YP, Mason S, Krishna LK. Enhancing geriatric oncology training through a combination of novice mentoring and peer and near-peer mentoring: A thematic analysis of mentoring in medicine between 2000 and 2017. Journal of geriatric oncology. 2019.
