# Supplementary material for: Mentoring future mentors in undergraduate medical education
Source: PLoS One. 2022 Sep 15;17(9):e0273358. doi: 10.1371/journal.pone.0273358 (PMC9477267; doi:10.1371/journal.pone.0273358)
Supplement: S2 Appendix — (DOCX) [file pone.0273358.s002.docx]

**SUPPLEMENTARY INFORMATION**

**S2 Appendix. Post-interview Survey Questions**

|  | **Responses (n; %)** | | | | |
| --- | --- | --- | --- | --- | --- |
| **Survey Item** | **1** | **2** | **3** | **4** | **5** |
| My reason to near peer mentor is to help my mentees develop into their own individual person. | 0, 0% | 0, 0% | 0, 0% | 8, 72.7% | 3, 27.3% |
| My reason to near peer mentor is to help my mentees optimize their wellbeing. | 0, 0% | 0, 0% | 0, 0% | 6, 54.5% | 5, 45.5% |
| My reason to near peer mentor is to help my mentees become better learners. | 0, 0% | 0, 0% | 0, 0% | 6, 54.5% | 5, 45.5% |
| My reason to near peer mentor is to help my mentees envision what kind of professional they want to be in the future. | 0, 0% | 0, 0% | 3, 27.3% | 5, 45. 5% | 3, 27.3% |
| As a near peer mentor, I am a sort of "help desk" for my students, providing them with information or referring them to resources. | 0, 0% | 0, 0% | 0, 0% | 3, 27.3% | 8, 72.7% |
| As a near peer mentor, I provide my mentees with insights into how the academic world works. | 0, 0% | 0, 0% | 1, 9.09% | 8, 72.7% | 2, 18.2% |
| As a near peer mentor, I help my mentees gain better understanding of the results of their actions. | 0, 0% | 0, 0% | 1, 9.09% | 5, 45. 5% | 5, 45. 5% |
| As a near peer mentor, I am my mentees' trusted person within the program. | 0, 0% | 0, 0% | 0, 0% | 8, 72.7% | 3, 27.3% |
| For me as near peer mentor, the personal development of my mentee is extremely important. | 0, 0% | 0, 0% | 0, 0% | 3, 27.3% | 8, 72.7% |
| For me as near peer mentor, having access to progress indicators of my mentee is critical. | 0, 0% | 0, 0% | 1, 9.09% | 7, 63.6% | 3, 27.3% |
| It is my mentees' own responsibility to ask me for advice if they have any questions. | 1, 9.09% | 1, 9.09% | 3, 27.3% | 6, 54.5% | 0, 0% |
| I advise my mentees what they should do based on my own experiences. | 0, 0% | 0, 0% | 0, 0% | 4, 36.4% | 7, 63.6% |
| I cannot solve problems for my mentees, they have to do that themselves. | 0, 0% | 4, 36.4% | 7, 63.6% | 0, 0% | 0, 0% |
| I can help my mentees to solve problems. | 0, 0% | 0, 0% | 2, 18.2% | 6, 54.5% | 3, 27.3% |
| If my mentees fail to meet expected performance standards, I will let them know. | 0, 0% | 1, 9.09% | 0, 0% | 5, 45. 5% | 5, 45. 5% |
| If my mentees want feedback on how they are doing, they should ask me for it. | 0, 0% | 3, 27.3% | 2, 18.2% | 4, 36.4% | 2, 18.2% |
| I want my mentees to adhere to my professional norms. | 0, 0% | 0, 0% | 1, 9.09% | 10, 90.9% | 0, 0% |
| My relationship with my mentees is based on an equal power balance. | 0, 0% | 0, 0% | 1, 9.09% | 4, 36.4% | 6, 54.5% |
| The amount of support I provide depends on the needs of each of my mentees. | 0, 0% | 0, 0% | 0, 0% | 5, 45. 5% | 6, 54.5% |
| There is a limit to the amount of support I am prepared to give to my mentees. | 0, 0% | 2, 18.2% | 0, 0% | 7, 63.6% | 2, 18.2% |

*Legend: 1 = Completely untrue for me, 2 = Somewhat untrue for me; 3 = Neither true nor untrue for me; 4 = Somewhat true for me; 5 = Completely true for me*
